# Detailed Urban Land Use Land Cover Classification at the Metropolitan Scale Using a Three-Layer Classification Scheme

**DOI:** 10.3390/s19143120

**Published:** 2019-07-15

**Authors:** Guoyin Cai, Huiqun Ren, Liuzhong Yang, Ning Zhang, Mingyi Du, Changshan Wu

**Affiliations:** 1 School of Geomatics and Urban Spatial Informatics, Beijing University of Civil Engineering and Architecture, Beijing 100044, China; 2 Beijing advanced innovation center for future urban design, Beijing University of Civil Engineering and Architecture, Beijing 100044, China; 3 Remote Sensing Application Center, Ministry of Housing and Urban-Rural Development of the People’s Republic of China, Beijing 100835, China; 4 Institute of Geographic Sciences and Natural Resources Research, Chinese Academy of Sciences, Beijing 101408, China; 5 Department of Geography, University of Wisconsin-Milwaukee, Milwaukee, WI 53211, USA

**Keywords:** urban land use/land cover, three-layer classification scheme, GF-1 satellite imagery, GF-2 satelliteimagery

## Abstract

Urban Land Use/Land Cover (LULC) information is essential for urban and environmental management. It is, however, very difficult to automatically extract detailed urban LULC information from remote sensing imagery, especially for a large urban area. Medium resolution imagery, such as Landsat Thematic Mapper (TM) data, cannot uncover detailed LULC information. Further, very high resolution (VHR) satellite imagery, such as IKONOS and QuickBird data, can only be applied to a small area, largely due to the data unavailability and high computation cost. As a result, little research has been conducted to extract detailed urban LULC information for a large urban area. This study, therefore, developed a three-layer classification scheme for deriving detailedurban LULC information by integrating newly launched Chinese GF-1 (medium resolution) and GF-2 (very high resolution) satellite imagery and synthetically incorporating geometry, texture, and spectral information through multi-resolution image segmentation and object-based image classification (OBIA). Homogeneous urban LULC types such as water bodies or large areas of vegetation could be derived from GF-1 imagery with 16 m and 8 m spatial resolutions, while heterogeneous urban LULC types such as industrial buildings, residential buildings, and roads could be extracted from GF-2 imagery with 3.2 m and 0.8 m spatial resolutions. The multi-resolution segmentation method and a random forest algorithm were employed to perform image segmentation and object-based image classification, respectively. An analysis of the results suggests an overall accuracy of 0.89 and 0.87 were achieved for the second and third level urban LULC classification maps, respectively. Therefore, the three-layer classification scheme has the potential to derive high accuracy urban LULC information through integrating medium and high-resolution remote sensing imagery.

## 1. Introduction

Land Use/Land Cover (LULC) is defined as the physical composition and characteristics (e.g., grass, forest, and impervious surfaces) or human-related activities (e.g., residential, commercial, and transportation) of land elements on the Earth’s surface [1]. The Climate Research Committee of the National Council stressed that the distribution of LULCs has a pronounced impact on Earth’s radiation balancing, since any changes in LULC would affect evaporation, transpiration, and heat flux on the ground surface [2]. Therefore, it is important for scientists and practitioners to understand LULC patterns and to monitor the changing world from global to local scales [3]. Satellite remote sensing has been demonstrated to be the most economic, efficient, and reliable data source for deriving LULC maps [4]. Currently, national and international agencies have successfully created no less than ten global scale LULC datasets with spatial resolutions of 1 km, 500 m, 300 m, 30 m, and 12 m. These existing LULC datasets provide basic geographic information for studying climate, hydrology, environment, ecology, and urban regions [5,6]. With the exception of the Global Urban Footprint, which is mainly used for mapping human settlements, all other LULC datasets are not specifically for urban LULC mapping. Four major urban LULC classes in the National Land Cover Database (NLCD) are developed open space, low intensity, medium intensity, and high intensity [7]. Another urban LULC class, artificial surfaces, is in the Globaland30 dataset [8], while the Global Land Cover 2000 (GLC-2000) product contains an urban LULC class for artificial surfaces and associated areas. There is also an LULC type for urban/built-up areas in the International Geosphere-Biosphere Programme (IGBP) classification scheme [9].

Complementary to the medium resolution imagery, very high resolution (VHR) optical satellite sensors provide remote sensing imagery with a sub-meter pixel resolution and detailed earth surface information [10]. Even with problems such as high image cost, shadowing effect, and relief displacement, fine spatial resolution imagery has emerged as an essential source to derive detailed urban LULC maps [11]. Many meter-level or sub-meter level spatial resolution sensors, such as IKONOS, OrbView, QuickBird, and WorldView, allow accurate mapping of LULC classes in urban and surrounding areas [12,13,14,15]. The applications of VHR-derived urban LULC information mainly focus on monitoring subtle changes, detecting urban villages in mega cities, and delineating tree crowns [16,17]. Because of the high complexity and heterogeneity, almost all urban LULC extractions from fine spatial resolution imagery are based on object-oriented classification algorithms and cover areas of less than one hundred square kilometers with emphasis on classification algorithm development and validation [18,19]. The urban LULC classes employed in these studies only represent several commonly used land uses or land covers rather than detailed urban classes.

Many metropolitan scale studies, such as dynamic urban growth analysis, detection of urbanization, and monitoring of urban heat islands, are all based on Landsat imagery [20,21,22]. Although integrating VHR image and LiDAR data is another means to map urban LULC for a large area, it faces problems of data shortage, high cost, small footprint, and large data volume [23,24]. The newly launched high-resolution Chinese satellite GF-1, with 16 m, 8 m, and 2 m spatial resolutions, is characterized by a wide swath and high resolution, and GF-2 is the first Chinese satellite with a spatial resolution lower than 1 m. These two satellites provide an opportunity to derive large areas of city-scale urban LULC information by combining multi-scale resolution images and designing multi-level classification schemes. By designing a three-layer classification scheme specifically for urban planning, this study expands the potential applications of satellite-derived urban LULC types to the relevant fields of urban planning, construction, and management. This can be implemented by measuring the industrialized production of urban sizes over recent decades to estimate the process of urbanization at a national level, or by providing basic geometric information on urban LULC components in residential or industrial regions. As is, these applications are limited by many factors, including coarser spatial resolution, limited urban LULC types in existing LULC products, and the lack of urban planning and management-specific classification schemes. Thus, it is difficult to apply the classified urban LULC maps to urban planning or management applications. The goal of this study, therefore, is to: (1) develop a three-layer urban specific LULC classification scheme; and (2) attempt to derive high accuracy urban LULC maps while minimizing fieldwork investigations and post-processing procedures to keep potential urban-related applications both cost effective and operationally practical.

The remainder of this paper is organized as follows: Section 2 introduces the methodology including study area, data sources, data processing, classification scheme, and methods for accuracy assessment; Section 3 details the results; Section 4 provides discussion; and Section 5 concludes the paper.

## 2. Methodology

### 2.1. Study Area

**Figure 1:**
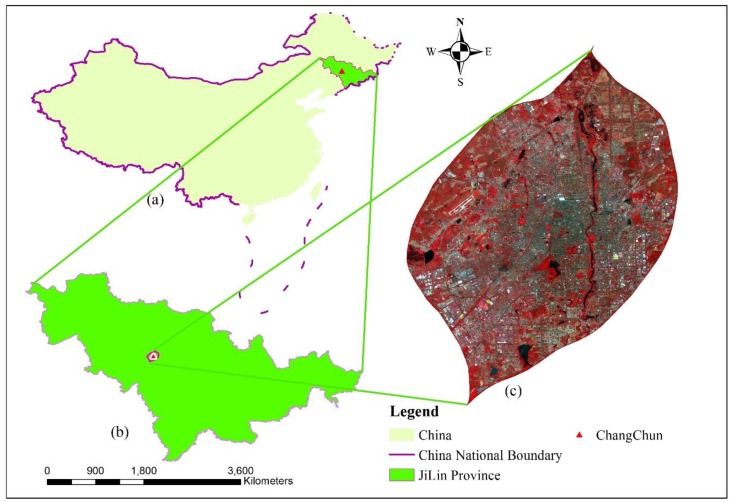
The study area (standard false color composition from GF-1 satellite imagery).

Changchun, the capital city of Jilin province, is located in the Northeast of China covering a region of longitudes from 124°18′ East to 127°05′ East and latitudes from 43°05′ North to 43°15′ North. It belongs to a temperate continental monsoon climate zone with an average temperature of 4.8 °C and an annual precipitation of 522 mm to 615 mm. Changchun is consisted of seven districts and three counties with a total governmental area of 20,604 km^2^ and population of 7.793 million including 4.509 million registered citizens in Changchun city. The area enclosed by the expressway surrounding the urban region is selected as the study site with an area of 523.16 km^2^ in this research (Figure 1).

### 2.2. Data and Data Processing

#### 2.2.1. Satellite Data

The Chinese GF-1 is the first satellite of the China High-resolution Earth Observation System. The GF-1 satellite was launched in April 2013 with two panchromatic/multi-spectral (P/MS) and four wide field view (WFV) cameras. GF-1 P/MS data have a spatial resolution of 2 m/8 m and swath width of 60 km, while WFV data have a spatial resolution of 16 m and swath width of 800 km with four spectral channels, which are highly valuable data sources for estimating fractional vegetation cover, building density, and monitoring suspended particulate matter on a large extension [25,26,27].

The Chinese GF-2 was launched after one and half year of the successful operation of the GF-1. The GF-2 is the first satellite with a spatial resolution lower than one meter which marks the China’s civil satellite enterprise into an era of sub-meter spatial resolution. The GF-2 is equipped with two fine resolution 0.8 m panchromatic, 3.2 m multi-spectral cameras, and a swath width of 45.7 km. It is featured as finer spatial resolution, high position accuracy, and fast maneuverability [28]. The detailed sensor characteristics for GF-1 and GF-2 are presented in Table 1.

The GF-1 imagery on June 22, 2015 and GF-2 imagery on May 25, 2015 were collected to conduct this study. Because of the large areas in this study area, six swaths of imagery were employed and their characteristics are listed in Table 2.

#### 2.2.2. In Situ Data Collection

**Figure 2:**
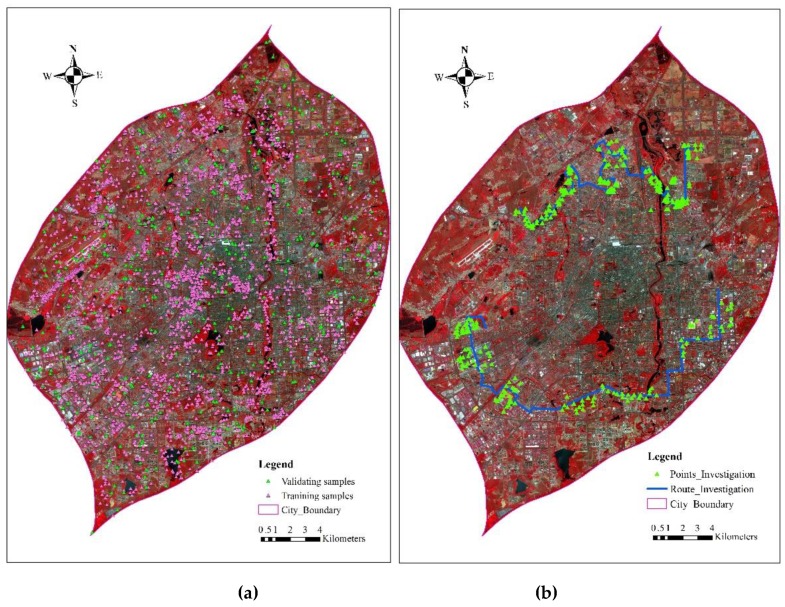
Land use land cover samples collection and field investigation, (**a**) collected training and testing samples; (**b**) field work route and investigation sites.

Based on the visual analysis and interpretation of GF-2 and GF-1 false color images (with bands four, three, and two as RGB for display) and the prior knowledge of this region, we identified and labelled 21 urban LULC types (Table 3), including two types of water bodies, two types of vegetation, one type of farmland, two types of bare lands, two types of roads and squares, five types of industrial buildings, and seven types of residential buildings, as well as shadow. The choices of these 21 LULC types are based on the practices of the Ministry of Housing and Urban-Rural Development of China, as well as the capability of visual interpretation from the 0.8 m resolution GF-2 imagery. Shadow is not an urban LULC type, but it is listed here as it cannot be grouped into other types. More than 100 points for each LULC type and altogether 2,732 points were manually collected from the image (Figure 2a). These samples were selected to ensure the coverage of all available urban land use land covers. While most of the samples have be successfully identified using GF-1 and GF-2 images, few of them are, as yet, unidentified. To address this issue, a field investigation was performed in Changchun city in June 2017 (Figure 2b) when the season coincides with that of the employed satellite imagery. A fieldwork route with 321 points was planned out on the image and imported into two GPS receivers whose positioning accuracy is 0.2–0.5 m. The current LULC information at each site was checked and photos were taken at the same time. Based on the field work, the wrongly interpreted LULCs were corrected in the laboratory and regarded as the training points. At the same time, the field work is helpful to interpret the testing points for accuracy assessment.

#### 2.2.3. Data Processing

**Figure 3:**
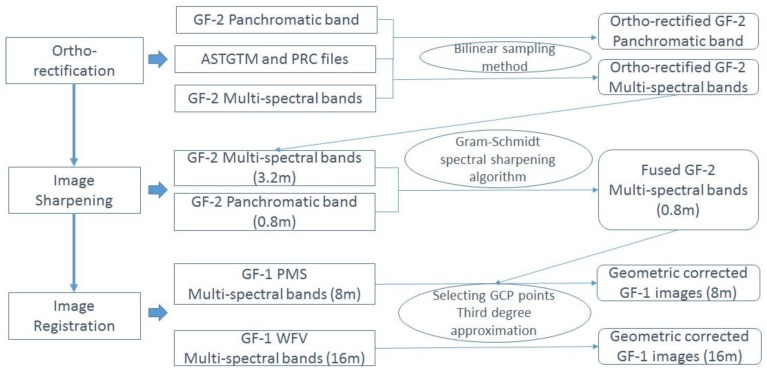
Flowchart of image pre-processing.

All of the images were transformed into the same spatial coordinate system of UTM zone 51N with datum of WGS 84. With the help of DEM dataset of ASTGTM (http://datamirror.csdb.cn) and the rational polynomial coefficients (RPC) file [29], the multispectral, and panchromatic images were ortho-rectified. The Gram-Schmidt spectral sharpening algorithm [30,31] was employed to fuse the ortho-rectified multispectral image (as the low resolution image) and the ortho-rectified panchromatic image (as the high resolution image), such that the fused multi-spectral imagery can be with a high spatial resolution of 0.8 m. The Gram-Schmidt spectral sharpening algorithm is a classic technique for image fusion, and relevant studies have shown its advantages over other methods [30,31]. In this research, the Gram-Schmidt algorithm embedded in ENVI, a commercial program, was adopted. As a result, all four bands of the GF-2 multi-spectral imagery were merged to generate the pansharpened imagery with four bands and 0.8 m resolution. As geometric differences exist between GF-1 and GF-2 images, GF-1 images were rectified with an RMS of 0.02 GF-1 pixel by selecting ground control points (GCPs) from the fused GF-2 image. Finally, we obtained four levels of imagery with spatial resolutions of 16 m, 8 m, 3.2 m, and 0.8 m, respectively. The flowchart for image pre-processing is presented in Figure 3.

### 2.3. Classification Scheme

Finer resolution images can provide more detailed information, which might be helpful to city managers and plannersfor extracting meaningful LULC types. Based on visually interpreted spectral differences, field investigation, and possible applications to urban planning and management, a three-layer classification scheme of urban LULC types was developed and is illustrated in Table 3.

The design of this three-layer land cover scheme is mainly based on the possible uses of land cover in urban-related applications, like urban planning and management. For instance, a city suffering from rapid urbanization needs urgent green space planning because the high rate of population growth contributes to diminishing green space. Detailed land cover information about grass, trees, and shrubs should be identified before making a reasonable plan for green space, while the land cover information of vegetation is adequate for a project on urban landscape change analysis. In the three-layer classification scheme designed here, water/pervious, impervious, and shadow land covers belong to the first level of land cover types. Water pervious and impervious land covers are critical parameters for urban hydrology, ecology, or environmental studies. With the objective of extracting the visually recognizable land cover types from GF-1 and GF-2 imagery, the second and third levels of urban LULC classification scheme were designed. In particular, buildings with different land uses present different spectral, textural, or geometric features, which lead to the possibility of separating LULC types of buildings in as much detail as possible. Considering the fact that a planning agency official conducting a land use or land cover inventory may wish to map several different types of roofs rather than a single building class. Building roofs are not only described as the last defining touch to giving a building, the aesthetic impression to a whole construction process, but also as expression and sign of a society’s level of civilization [32,33]. In addition, building roofs, especially with red or white colors are usually used to identify industrial or logistical land use information in regions surrounding central urban areas. Five types of industrial buildings, five types of residential buildings, as well as playgrounds were identified visually by geometric and spectral features.This process has been carried out mainly by visual interpretation of images, and is then corrected by in situ investigation and validation. Some land cover types in the third layer belong to the same land cover or land use: For instance, the residential or industrial buildings types. Although they are nearly meaningless for urban planning or management projects, they are helpful in extracting the second layer of the urban LULC types with high accuracy or beneficial to detect roofing materials. In practice, particularly in China, buildings with the same use are likely to be with the same or similar color. It is more likely to be a planning practice in China.

### 2.4. Image Segmentation and Classification

For segmentating and classifying GF-1 and GF-2 imagery, an object-based approach was employed in this study. Object-based image analysis processing generally includes two main steps: Segmentation and classification [34]. The first step in the object-oriented image classification is to segment the image into different objects. Numerous image segmentation algorithms have been developed and applied in remote sensing image analysis [35]. Based on the multi-level classification scheme designed in this research, the algorithm of multi-resolution segmentation (MRS) was selected as the segmentation method [36]. The MRS technique is a region-merging method. Its objective is to minimize the summed heterogeneity between adjacent pixels. Three user-defined segmentation parameters, including scale, shape, and compactness, could have a significant effect on the classification accuracy as they control the dimension and size of segmented objects [37,38]. Scale, the most important parameter, specifies the size of the final segmented image object that corresponds to the maximum acceptable heterogeneity. Higher scale parameter values produce larger image objects and vice versa. The shape parameter varies between zero and one and determines both the level of radiometric homogeneity and object shape, simultaneously. Higher shape values yield image objects with optimal shape homogeneity, while lower shape values produce image objects with optimal radiometric homogeneity. Same as the parameter of shape, the compactness parameter varies between zero and one and controls the degree of object smoothing [39]. These three user-defined parameters are affected by different image spatial resolutions and the sizes of the recognized ground objects [40]. Based on research from Drăgut et al., in 2010 and 2014 [41,42], the optimal segmentation scale parameters for images with spatial resolutions of 16 m and 8 m were designed as 100, while for images with spatial resolutions of 3.2 m and 0.8 m were 50 and 25, respectively. The detailed segmentation parameters could be found in Table 4.

Classifiers like decision tree (DT), random forest (RF), and support vector machine (SVM) have attracted great attention among many object-oriented classification algorithms owing to their excellent classification performance [43,44]. For medium spatial resolution images, the performances of these three algorithms are similar [45]. For classifying object-based imagery with finer spatial resolution, inconsistent conclusions have been drawn due to the effect of various factors such as segmentation parameters [46,47,48]. By systematically analyzing the performance of various commonly-used supervised classifiers under different conditions, Li et al. (2016) concluded that RF was most suitable supervised classification method for object-based image analysis [49].

RF is an ensemble classification technique and is a further development of DTs [50]. RF has advantages over DT due to its characteristics of little training time, easy parameterization, and parameter stability. Therefore, RF has attracted more attentions around the scientific community. Unlike DT classifiers, RF runs iteratively with a random sample of the training points,and because of the law of large numbers, RF reduces the likelihood of over-fitting [51]. In addition, compared with other commonly used non-parametric classifiers such as SVM, RF is less sensitive to noise and is more efficient [52]. Due to its advantages, RF was employed in this research with urban LULC types as the dependent variable, and spectral features, i.e., mean values of each individual band, band composition of normalized difference vegetation index (NDVI), geometric information of shape index, texture measures, i.e., homogeneity, angular second moment, contrast, and entropy from the grayscale co-occurrence matrix (GLCM) which were calculated in a sliding window of 11 by 11 pixels, as the main input features [53,54]. The processes of image segmentation, classification and the following section of accuracy assessment were carried out through eCognition developer 9.0.

### 2.5. Urban LULC Type Extraction

**Figure 4:**
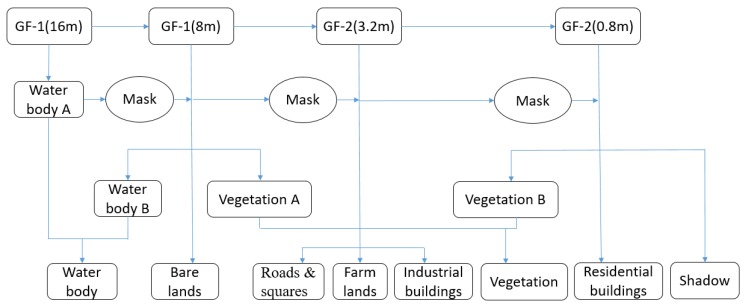
Flowchart of information combination from GF-1 and GF-2 multi-resolution satellite imagery.

A three-layer classification scheme was designed for this studyto identify different urban LULC types from GF-1 and GF-2 imagery with different spatial resolutions. This multi-level classification can provide city planners with an approach for selecting appropriate LULC types by combining or separating these extracted land use/land covers. In contrast to LULC classes like buildings, squares, and gardens within residential regions, LULCs like water bodies, bare lands, and urban green lands have a large area of existence such that they could be identified by GF-1 data with a 16 m spatial resolution. After masking out those extracted LULCs, those with small areas could be classified using finer resolution images. During each step of LULC classification, the minimum mapping unit (MMU), which is based on the MMU values from Globeland30 products, is employed to extract the LULC types (Table 4) [8]. Because of spatial resolution differences among images employed in this research, there are some inconsistencies in the boundaries of each type of LULC. In order to avoid slivers caused by the inconsistencies, we generated polygon vector files for regions whose LULC information has been extracted from GF-1 images, those regions were not processed any further. Only regions outside these vector files are classified from GF-2 images. These vector files along with raster files were regarded as the input data for the next step urban LULC extraction. In addition, shadow does not belong to any type of LULC, but it is widely present in VHR images, especially for these covering urban areas or mountainous regions, so shadow is presented as an individual type. The combination of the information from GF-1 and GF-2 satellite images with different spatial resolutions for extracting each urban LULC type is illustrated in Figure 4.

#### 2.5.1. Water Body

**Figure 5:**
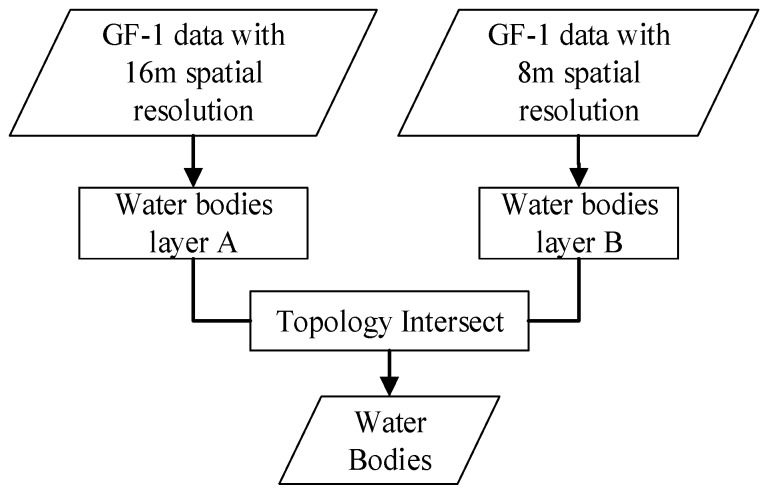
Flowchart for extracting water bodies.

There are two types of water bodies identifiable from the visual image interpretation: One is clean water while the other is turbid water. They are classified individually and then merged together and termed the water body class. Because of the relatively large areas of water bodies, the LULC information for water bodies were classified from the GF-1 data with 16 m and 8 m spatial resolutions. Because the effect of shadows on the identification of water bodies was much heavier in images with an 8 m spatial resolution than in images with a 16 m spatial resolution, an overlay spatial analysis was employed to perform topology intersect operations between water bodies extracted from images at each spatial resolution. The intersect operation is an “AND” logical operation, and the resultant polygon is classified as water bodies if both inputs are classified as water bodies. The overlying results are considered to be the main part of water bodies. The detailed workflow for extracting water bodies is shown in Figure 5.

#### 2.5.2. Vegetation

Vegetation is mainly composed of trees, shrubs, and grass. The spectral information from grass is different from that of trees and shrubs, so they can be separately extracted and then merged together as vegetation. Large areas of vegetation such as urban gardens and parks can be extracted from GF-1 images with an 8 m spatial resolution, while small areas of vegetation in residential regions can be identified from GF-2 images with a 0.8 m spatial resolution.

#### 2.5.3. Bare Lands

**Figure 6:**
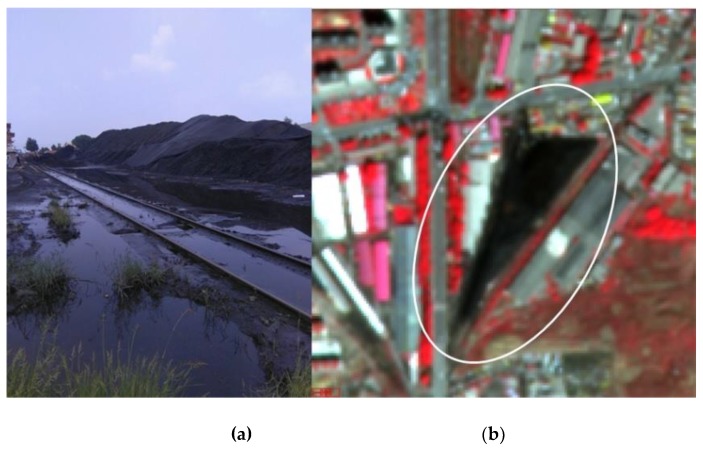
(**a**) a coal ash site and (**b**) its appearance in the image.

**Figure 7:**
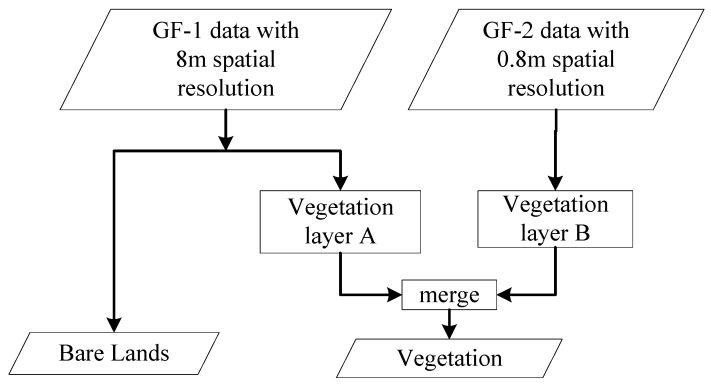
Flowchart for extracting vegetation and barren lands.

Most of the bare lands are located in regions surrounding urban areas. They are mainly construction sites. Additionally, there are places with piles of coal ash, and we classified them as bare lands as well. This type of land cover is very difficult to be identified from visual interpretation of the images. Therefore, they have been further investigated through field work and shown in Figure 6. Because bare lands are relatively large and are unlikely to be misclassified as other land covers, they were extracted from low resolution images (e.g., GF-1 imagery with 8 m spatial resolution). The flow chart for extracting vegetation and bare lands is shown in Figure 7.

#### 2.5.4. Farm Lands, Roads and Squares, and Buildings

For this research, GF-1 images were collected in summer, while GF-2 images were acquired in late spring. During the late spring, many farm lands only contain bare soil with scattered crops, while trees, shrubs, and grass are with green leaves. Therefore, it is feasible to distinguish farmlands from vegetation (e.g., trees, shrubs, and grass) using GF-2 data. Because most farm lands are with regular geometric patterns and occupy large geographic areas, GF-2 data with a 3.2 m spatial resolution were employed to classify farm lands.

In addition to farm lands, roads were extracted from GF-2 images with a 3.2 m spatial resolution. Roads were characterized as linear features and squares were characterized by very bright tones. Because it is difficult to differentiate between roads and buildings from the classified images, GPS trace data of the main roads were employed to identify roads by topological intersection with the classified LULC map. All roads intersecting with GPS trace data were selected and regarded as the final data for the main roads, while others were categorized as buildings.

**Figure 8:**
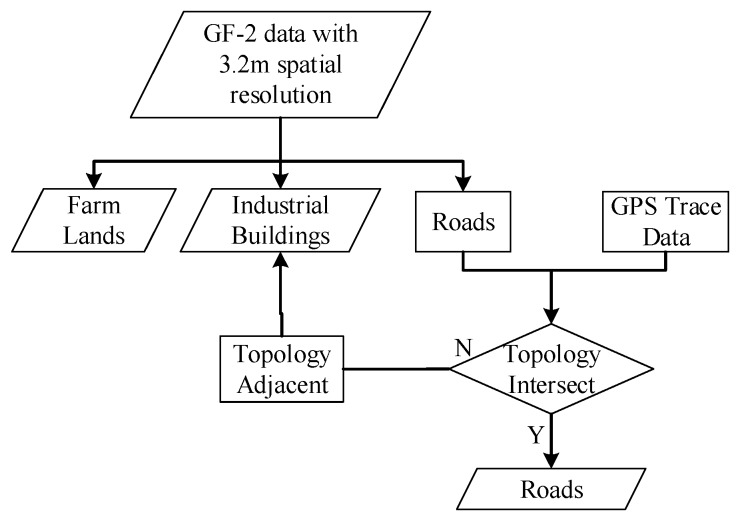
Flowchart for extracting farm lands, roads and squares, and buildings.

Changchun is a typical automobile city with many relatively large industrial buildings compared to residential buildings. GF-2 imagery with a 3.2 m spatial resolution was used to extract industrialbuildings, while imagery with a 0.8 m spatial resolution was employed to extract residential buildings and small sized shadows (Table 4). The flowchart for extracting farm lands, roads and squares, and buildings is shown in Figure 8.

### 2.6. Accuracy Assessment

Because of the advantages in making sure samples will be included in each class, we employed the stratified random sampling protocol to collect the testing points. Pixels were used as the spatial unit in this work. All of the testing points were visually interpreted by overlying on GF-2 false color composition image or on GF-1 image for areas without the coverage of GF-2 image. Pixel based error matrix was employed to compute the overall accuracy (O), user’s accuracy (U), and producer’s accuracy (P), and to quantitatively assess the accuracy of the urban LULC map [55,56]. In the error matrix, *p_ij_* represents the proportion of area for the population that has map class I and reference class j. Overall accuracy derived from an error matrix of q classes can be expressed as [57]:

(1) $$O=\sum_{j=1}^{q}p_{ij}$$

User’s accuracy of class i is:

(2) $$U=p_{ii}/p_{i\cdot }$$

And producer’s accuracy of class j is:

(3) $$P=p_{jj}/p_{\cdot j}$$

The cell entries of the population error matrix and the parameters derived from it must be estimated from a sample. The sample based estimator of *p_ij_* is denoted as ${\hat{p}}_{ij}$, and correspondingly, the error matrix should be reported in terms of these estimated area proportions ${\hat{p}}_{ij}$ instead of sample counts, n*_ij_*. ${\hat{p}}_{ij}$ can be expressed as:

(4) $${\hat{p}}_{ij}=W_{i}\frac{n_{ij}}{n_{i\cdot }}$$

where *W_i_* is the proportion of area mapped as class *i*. replacing *p_ij_* by ${\hat{p}}_{ij}$, we can calculate the overall, user’s and producer’s accuracies.

Aside from accuracy parameters, standard errors should be reported to indicate the sampling variability. For user’s accuracy of map class *i*, the estimated variance is:

(5) $$\hat{V}\left({\hat{U}}_{i}\right)={\hat{U}}_{i}\left(1-{\hat{U}}_{i}\right)/\left(n_{i\cdot }-1\right))$$

For producer’s accuracy of reference class j=k, the estimated variance is:

(6) $$\hat{V}\left({\hat{P}}_{j}\right)=\frac{1}{{\hat{N}}_{\cdot j}^{2}}\left[\frac{N_{j\cdot }^{2}{\left(1-{\hat{P}}_{j}\right)}^{2}{\hat{U}}_{j}\left(1-{\hat{U}}_{j}\right)}{n_{j\cdot }-1}+{\hat{P}}_{j}^{2}\sum_{i\neq j}^{q}N_{i\cdot }^{2}\frac{n_{ij}}{n_{i\cdot }}\left(1-\frac{n_{ij}}{n_{i\cdot }}\right)/\left(n_{i\cdot }-1\right)\right]$$

where $N_{\cdot j}=\sum_{i=1}^{q}\frac{N_{i\cdot }}{n_{i\cdot }}n_{ij}$ is the estimated marginal total number of pixels of reference class *j*, $N_{j\cdot }$ is the marginal total of map class *j* and $n_{j\cdot }$ is the total number of sample units in map class *j*.

In addition, error matrix provides the basis for estimating the areas of classes. If the sampling design is simple random, systematic or stratified random, an estimator of the proportion of areas of class *k* is:

(7) $${\hat{p}}_{\cdot k}=\sum_{i=1}^{q}W_{i}\frac{n_{ik}}{n_{i\cdot }}$$

For the stratified estimator of proportion of area (Equation (7)), the standard error is estimated by:

(8) $$S\left({\hat{p}}_{\cdot k}\right)=\sqrt{\sum_{i}\frac{W_{i}{\hat{p}}_{ik}-{\hat{p}}_{ik}^{2}}{n_{i\cdot }-1}}$$

where *n_ik_* is the sample count at cell (*i*,*k*) in the error matrix, *W*_i_ is the area proportion of map class *i*, ${\hat{p}}_{\cdot k}=W_{i}\frac{n_{ik}}{n_{i\cdot }}$ and the summation is over the q classes. The estimated area of class *k* is ${\hat{A}}_{k}=A\times {\hat{p}}_{\cdot k}$, where *A* is the total map area.

The standard error of the estimated area is given by:

(9) $$S\left({\hat{A}}_{k}\right)=A\times S\left({\hat{p}}_{\cdot k}\right)$$

An approximate 95% confident interval is obtained as ${\hat{A}}_{k}\pm 1.96\times S\left({\hat{A}}_{k}\right)$.

## 3. Results

### 3.1. Urban LULC Mapping

**Figure 9:**
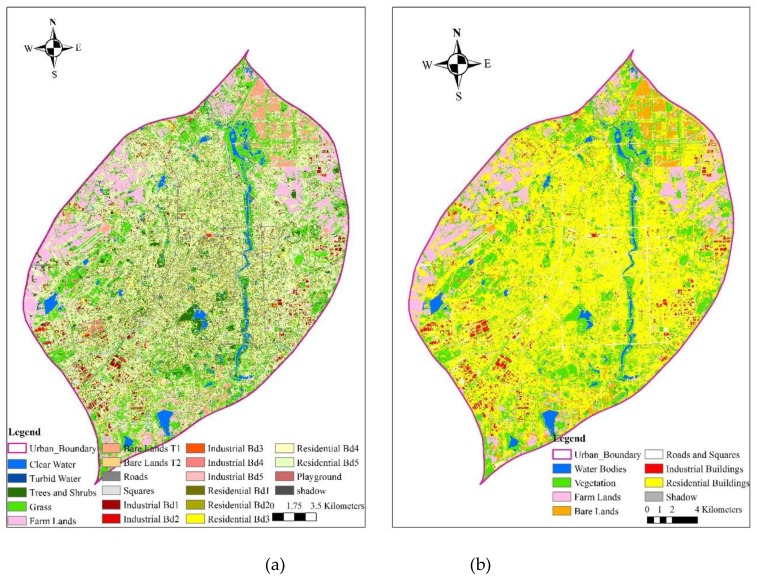
Urban LULC map of Changchun city: (**a**) the third layer, and (**b**) the second layer.

**Figure 10:**
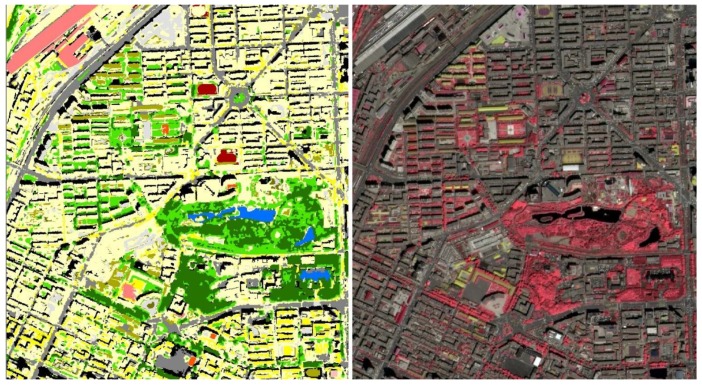
Part of the zoomed view of the LULC layer 3 map (left) and the corresponding GF-2 standard false color composition image (right) in the central urban region of Changchun City.

Based on the above mentioned method, urban LULC maps of classification scheme layers two and three were obtained through applying the object-based RF classifiers and are presented in Figure 9. As can be discerned from Figure 9, the third layer of the urban LULC map (Figure 9a) presents more detailed urban land cover information when compared with the second layer with eight urban LULC types (Figure 9b). Compared with the third layer, the second layer of the urban LULC map looks more consistent with the general impression. That is, the central urban area is covered by residential buildings, green lands, and water bodies, while the surrounding regions are covered by industrial buildings, farm lands, and construction lands. For a better visualization, a portion of third layer of the urban LULC map was zoomed in, and visualized compared with the GF-2 image (Figure 10). Similarly, it indicates a good consistency exists between the classified urban LULC types and the GF-2 image.

### 3.2. Quantitative Accuracy Assessment

Accuracy assessment determines the quality of a classified map from satellite images. In this paper, we performed the process of accuracy assessment according to the good practices for estimating accuracy recommended by Olofsson et al. (2014) [57].

#### 3.2.1. Estimating Accuracy

The estimation of map accuracy is conducted by a plugin named “AcATaMa” version 18.11.21 and installed in QGIS 3.4.4. Stratified random sampling approach was used, sample sizes were calculated and allocated according to the mapped proportion of area of each class. Because of the uneven of the mapped area of classes, some classes with small areas were allocated with only five or even less samples. For being more representative of the samples in each class, we manually adjusted samples with a minimum number of 10. All of the testing points were labeled by two different analysts together. Finally, the urban LULC maps layer two and layer three error matrices resulting from the sample and response design are presented in terms of the sample counts displaced in Table 5 and Table A1. In which, user’s accuracy, producer’s accuracy, and overall accuracy for each class are presented. Table 5 shows that the overall accuracy of the second layer of the urban LULC map reaches 0.89, which means that the second layer of the urban LULC map is more accurate than the third layer of the urban LULC map whose overall accuracy is 0.87. Based on Equations (4)–(6), we can compute the estimated user’s and producer’s accuracy and variances by using an error matrices with cell values ofthe estimated area proportions (Table 6 and Table A2). The estimated user’s and producer’s accuracy with a 95% confident interval for urban LULC maps layer two and layer three are presented in Table 7. Although good overall accuracies are achieved for both layer two and layer three map classification scheme, there are some map classes, especially in map class layer three, with lower accuracies either in user’s or producer’s accuracy, for instance, map classes of bare lands T2, residential bd2–bd4, their accuracies are less than 0.80.

#### 3.2.2. Estimating Area and Uncertainty

Based on the estimated area proportions we can estimate the area of each class according to the reference data. For instance, the error matrix in Table 6 can be used to indicate how to estimate the area and uncertainty. The estimated area of water body (Wb) is ${\hat{A}}_{1}={\hat{p}}_{\cdot 1}\times A_{tot}=0.02613\times 523.1622=13.66806\;{\mathrm{km}}^{2}$, the mapped area of water body of 14.09400 km^2^ was thus underestimated by 0.42594 km^2^. The confident interval for the area of each class can be estimated based on the method mentioned in Section 2.6. From Equation (8), $S\left({\hat{p}}_{\cdot i}\right)=0.00082$ and the standard error for the estimated area of water body is $S\left({\hat{A}}_{1}\right)=S\left({\hat{p}}_{\cdot i}\right)\times A_{tot}=0.00082\times 523.1622=0.42900\;{\mathrm{km}}^{2}$. The margin of error of the confidence interval is $1.96\times 0.429=0.84084\;{\mathrm{km}}^{2}$. Such that we estimated the area of water body with a 95% confident interval is $13.66806\pm 0.84084\;{\mathrm{km}}^{2}$, i.e., the lower and upper limit is 12.82722 km^2^ and 14.5089 km^2^, respectively. The area of each map class in layer two and layer three can be estimated in the same way. Table 8 presents the estimated areas of each class with the second digit after the decimal place in urban LULC layers two and three with a confident interval of 95%. These can provide useful information for urban planners to examine the uncertainties of each map class, such that they can make some decisions in protection of some land uses.

## 4. Discussion

Medium resolution satellite imagery lacks of the ability of providing detailed urban LULC information. On the contrary, sub-meter and meter-scale satellite imagery provide essential data sources to extract detailed land cover information, especially for urban regions with highly heterogeneous manmade materials. These VHR imagery, however, cannot cover an entire city and generally computationally expensive. In this study, medium resolution imagery (GF-1) and very high resolution imagery (GF-2) were integrated to map metropolitan-scale urban LULC types by taking advantage of the multi-level resolution imagery from these two satellites. The acquisition of multi-level resolution imagery provides a unique opportunity to classify urban LULC types in a large urban area at different spatial scales. Considering the fact that urban LULC types with a relatively large areas, such as water bodies, green lands, and bare lands can be classified by GF-1 images while small sized urban LULC types, like industrial and residential buildings, can be extracted by GF-2 data, this work proposed a three-layer classification scheme. This scheme is very flexible, allowing for urban planners and policy makers to select which level of urban LULC type might be more appropriate for their specific applications, including spatio-temporal dynamics of urbanization, suburbanization, dynamic land cover or land use change, urban landscape change analysis, and ecology conservation [58,59,60]. Although with many issues, including the unavailability of two or more satellite images, relatively complex image processing techniques, and the difficulty of selecting appropriate urban LULC types, this scheme provides a practical approach to extract metropolitan-scale urban LULC types at different level of details.

The design of the three-layer land cover scheme is mainly based on the visually separability among each urban LULC type aiming to achieve a better accuracy. The accuracy assessment shows that many urban LULC types have achieved high accuracies using the designed three-layer classification scheme. Although there is not a definite threshold of accuracy for relative applications of urban LULC mapping from satellites, one goal of this study is to achieve the highest possible accuracy while minimizing fieldwork and post-processing procedures to keep potential applications both cost-effective and operationally practical. All of the urban LULC types in the second and third layer of the urban LULC map are appropriate for use by urban planners or managers to perform dynamic urban expansion analysis, to estimate urban population density and other urban landscapes and to conduct urban environment-related projects [61,62,63]. This scheme is specially designed for GF-1 and GF-2 satellite images. It may not perform well for other satellite images with different dimensions, but it can provide an approach to derive LULC types with different level of details. Most of the urban LULC types in this scheme are helpful for urban planners or local managers to understand the current conditions of urban LULCs.

## 5. Conclusions

More and more satellites with finer spatial resolutions have been successfully launched around the world in the last two decades. While finer scale imagery allows for differentiating more subtle geometric differencesin land cover types than coarse or medium spatial resolution images, problems such as the large volume of datasets and the length of time necessary for processing segmentation and classification still persist, especially for a large region like a whole urban area. By integrating GF-1 imagery with meter-resolution and GF-2 imagery with sub-meter-resolution, this study designed a three-level classification scheme and mapped detailed urban LULC at different spatial resolutions. Conclusions from a case study in Changchun, the capital city of Jilin Province, can be drawn as follows:

First, the proposed multi-level classification scheme is feasiblein extracting urban LULCs by combining medium resolution and VHR remote sensing imagery. Homogeneous urban LULC types such as water bodies, bare lands, or large areas of vegetation could be derived from GF-1 imagery with 16 m and 8 m spatial resolutions, while heterogeneous urban LULC types such as farm lands, industrial buildings, and roads and squares could be extracted from GF-2 imagery with 3.2 m spatial resolution, and residential buildings, small patches of vegetation, and shodows could be generated from GF-2 with 0.8 m spatial resolutions.

Second, through implementing the image segmentation and object-based image classification, detailed urban LULC maps at the second and third levels illustrate an overall accuracy of 0.89 and 0.87, suggesting the three-layer classification scheme has the potential to derive high accuracy urban LULC information.

## Figures and Tables

**Table 1:** Data characteristics for satellites GF-1 and GF-2.

Satellite	Spectral Bands	Spatial Resolution (m)	Spectral Range (μm)	Swath Width (km)
GF-1	P	2	0.45–0.90	
	MS	8	0.45–0.52	
			0.52–0.59	60
			0.63–0.69	
			0.77–0.89	
	WFV-MS	16	0.45–0.52 0.52–0.59 0.63–0.69 0.77–0.89	800
GF-2	P	0.8	0.45–0.90	
	MS	3.2	0.45–0.52	
			0.52–0.59	45.7
			0.63–0.69	
			0.77–0.89	

**Table 2:** Data used in this study.

Satellite	Image Number	Image Level	Acquiring Date	Spatial Resolution	Spectral Bands
GF-1	85656	1A	20150622	8 m/16 m	MS
	85657	1A	20150622	8 m/16 m	MS
	875773	1A	20150622	8 m/16 m	MS
	875774	1A	20150622	8 m/16 m	MS
GF-2	805806	1A	20150515	0.8 m/3.2 m	P/MS
	805807	1A	20150515	0.8 m/3.2 m	P/MS

**Table 3:** Three-level classification scheme for urban Land Use/Land Cover (LULC) extraction.

LULC Types			Images	Photos
Layer 1	Layer 2/Code	Layer 3/Code		
Pervious surfaces	Water body/1	Clear water/11	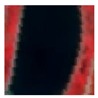	Rivers or lakes with dark tone without bathymetry information
		Turbid water/12	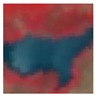	Water bodies with bright and light blue color with sediment information
	Vegetation/2	Trees and Shrubs/21	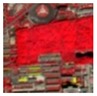	Densely planted trees or shrubs with shiny red color
		Grass/22	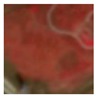	Densely planted grass with slightly dark red color
	Farm lands/3	Croplands/31	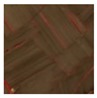	Exposed croplands with light brown color
Impervious surfaces	Bare lands/4	Construction sites without working on/41	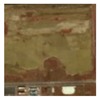	Exposed soil patches with light yellow color
		Coal ash site/42	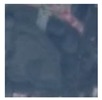	Exposed powdered coal with grey color
	Roads and squares/5	Roads/51	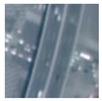	Urban roads with grey color
		Squares and airport runway/52	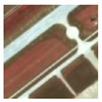	Aircraft runways with bright white color
	Industrial buildings/6	Red roofs/61	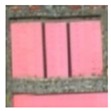	Industrial buildings with regular shape and bright red color
		Yellow roofs/62	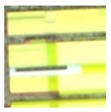	Industrial buildings with regular shape and bright yellow color
		White roofs/63	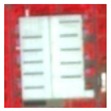	Industrial buildings with regular shape and bright white color
		Grey roofs/64	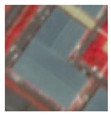	Industrial buildings with regular shape and grey color
		Purple roofs/65	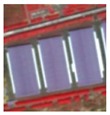	Industrial buildings with regular shape and purple color
	Residential buildings/7	Bright roofs/71	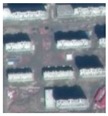	Residential low-rise buildings with relatively small sizes and bright white color
		Yellow roofs/72	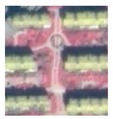	Residential low-rise buildings with relatively small sizes and yellow color
		Black roofs/73	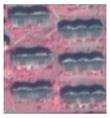	Residential low-rise buildings with relatively small sizes and dark color
		Red roofs/74	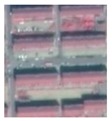	Residential low-rise buildings with relatively small size and grey color
		High-density white roofs/75	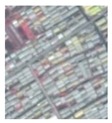	Intensive residential buildings with small size and grey color
		Playground/76	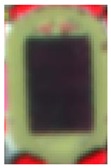	Playground with running track and rectangle-shape soccer field
Shadow	Shadow/8	Shadow/81	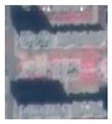	Shadow (adjacent to high-rise or low-rise buildings) with dark color

**Table 4:** Minimum mapping unit (MMU) and segmentation parameters for GF-1 and GF-2 satellites.

LULC Types	Satellite Imagery	Segmentation Parameters Scale/Shape/Compactness	Minimum Mapping Unit (MMU,pixel)
Water body A	GF-1(16 m)	100/0.4/0.5	3 × 3
Water body B	GF-1(8 m)	100/0.4/0.5	3 × 3
Vegetation A			10 × 10
Bare lands			6 × 6
Farm lands	GF-2(3.2 m)	50/0.4/0.5	6 × 6
Roads and squares			8 × 8
Industrial buildings			8 × 8
Vegetation B	GF-2(0.8 m)	25/0.6/0.5	100 × 100
Shadow			8 × 8
Residential buildings			8 × 8

**Table 5:** Assessment of the second layer of the urban LULC map.

	Sample	Wb	Vg	Fl	Bl	R&S	Ib	Rb	Sd	Tt	Ta (km^2^)	Wi
User Class												
Wb		32	0	0	0	0	0	0	1	33	14.10	0.03
Vg		0	63	1	2	0	0	1	1	68	109.29	0.21
Fl		0	0	62	8	0	0	2	0	72	53.07	0.10
Bl		0	0	0	42	0	0	0	0	42	18.09	0.03
R&S		0	0	0	1	41	3	1	0	46	20.04	0.04
Ib		0	0	0	0	0	31	0	0	31	13.57	0.03
Rb		0	4	1	6	3	4	55	2	75	288.19	0.55
Sd		0	0	0	0	0	1	2	27	30	6.81	0.01
Tt		32	67	64	59	44	39	61	31	397	523.16	0.03
Producer		1.00	0.94	0.97	0.71	0.93	0.79	0.90	0.87	1.00		
User		0.97	0.93	0.86	1.00	0.89	1.00	0.73	0.90	0.97		
Totals		Overall Accuracy 0.89										

**Notes:** Wb: Water bodies; Vg: Vegetation; Fl: Farm lands; Bl: Bare lands; R&S: roads and squares; Ib: Industrial buildings; Rb: Residential buildings. Sd: Shadow; Tt: Total; Ta: Total class area.

**Table 6:** The error matrix populated by estimated proportion of area for ULULC layer two.

	Sp	Wb	Vg	Fl	Bl	R&S	Ib	Rb	Sd	Wi
Uc										
Wb		0.03	-	-	-	-	-	-	0.00	0.03
Vg		-	0.19	0.00	0.01	-	-	0.00	0.00	0.21
Fl		-	-	0.09	0.01	-	-	0.00	-	0.10
Bl		-	-	-	0.03	-	-	-	-	0.03
R&S		-	-	-	0.00	0.03	0.00	0.00	-	0.04
Ib		-	-	-	-	-	0.03	-	-	0.03
Rb		-	0.03	0.01	0.04	0.02	0.03	0.40	0.01	0.55
Sd		-	-	-	-	-	0.00	0.00	0.01	0.01
Tt		0.03	0.22	0.10	0.10	0.06	0.06	0.41	0.03	

**Table 7:** The estimated user’s accuracy with a 95% confident.

Layer 2		Layer 3	
Urban LULC	Estimated Users’/Producers’ Accuracy	Urban LULC	Estimated Users’/Producers’ Accuracy
Water bodies	0.97 ± 0.06/1.00 ± 0.00	Clear water	0.90 ± 0.13/0.86 ± 0.17
		Turbid water	0.60 ± 0.26/1.00 ± 0.00
Vegetation	0.93 ± 0.06/0.94 ± 0.07	Trees and shrubs	1.00 ± 0.00/1.00 ± 0.00
		Grass	0.87 ± 0.14/0.83 ± 0.19
Farm land	0.86 ± 0.08/0.97 ± 0.09	Farm land	0.94 ± 0.12/0.84 ± 0.16
Bare lands	1.00 ± 0.00/0.71 ± 0.06	Bare lands T1	0.96 ± 0.88/0.92 ± 0.15
		Bare lands T2	1.00 ± 0.00/0.65 ± 0.11
Roads and squares	0.89 ± 0.09/0.93 ± 0.12	Roads	0.92 ± 0.11/0.82 ± 0.18
		Squares	0.93 ± 0.13/0.93 ± 0.15
Industrial buildings	1.00 ± 0.00/0.79 ± 0.06	Industrial Bd1	1.00 ± 0.00/0.88 ± 0.14
		Industrial Bd2	1.00 ± 0.00/0.88 ± 0.14
		Industrial Bd3	0.93 ± 0.13/0.93 ± 0.15
		Industrial Bd4	0.80 ± 0.21/1.00 ± 0.00
		Industrial Bd5	0.87 ± 0.18/0.87 ± 0.16
Residential Buildings	0.73 ± 0.10/0.90 ± 0.23	Residential Bd1	0.95 ± 0.07/0.93 ± 0.16
		Residential Bd2	0.73 ± 0.23/0.85 ± 0.17
		Residential Bd3	0.70 ± 0.18/0.95 ± 0.16
		Residential Bd4	0.73 ± 0.23/0.85 ± 0.17
		Residential Bd5	0.77 ± 0.13/0.76 ± 0.35
		Playground	0.80 ± 0.21/0.92 ± 0.15
Shadow	0.90 ± 0.11/0.87 ± 0.17	Shadow	0.80 ± 0.21/0.80 ± 0.19

**Table 8:** The estimated area (km^2^) of each class with a 95% confident.

Urban LULC	Area	Error	Lower Limit	Upper Limit	Urban LULC	Area	Error	Lower Limit	Upper Limit
Water bodies	13.67	0.43	12.83	14.51	Clear water	12.67	1.17	10.38	14.95
					Turbid water	0.59	0.13	0.34	0.84
Vegetation	116.63	8.30	100.37	132.88	Trees and shrubs	29.56	0.00	29.56	29.56
					Grass	84.88	10.21	64.87	104.89
Farm land	51.15	4.70	41.94	60.36	Farm land	58.46	6.83	45.08	71.85
Bare lands	50.69	9.58	31.92	69.47	Bare lands T1	20.98	5.03	11.13	30.84
					Bare lands T2	6.15	3.20	-0.12	12.42
Roads and Squares	29.39	6.63	16.39	42.39	Roads	31.61	8.52	14.92	48.30
					Squares	3.37	0.71	1.97	4.77
Industrial buildings	30.47	7.57	15.64	45.30	Industrial Bd1	8.04	0.81	6.45	9.64
					Industrial Bd2	2.88	0.39	2.11	3.65
					Industrial Bd3	2.10	0.24	1.64	2.56
					Industrial Bd4	1.61	0.21	1.19	2.03
					Industrial Bd5	5.83	5.02	−4.01	15.67
Residential Buildings	215.31	14.95	186.02	244.61	Residential Bd1	34.01	1.69	30.70	37.32
					Residential Bd2	4.87	0.99	2.94	6.80
					Residential Bd3	14.60	1.90	10.87	18.32
					Residential Bd4	6.60	1.06	4.52	8.67
					Residential Bd5	181.24	14.92	152.00	210.48
					Playground	1.36	0.47	0.44	2.28
Shadow	15.85	5.66	4.76	26.95	Shadow	11.75	5.12	1.72	21.78

## Appendix A

**Table A1:** Assessment of the land cover map in the third layer of the classification scheme.

S	Cw	Tw	Ts	Gs	Fl	Bl1	Bl2	Rd	Sq	Ib1	Ib2	Ib3	Ib4	Ib5	Rb1	Rb2	Rb3	Rb4	Rb5	Pg	Sd	Tt	TA (km^2^)	Wi
Cw	18	0	0	0	0	0	0	0	0	0	0	0	0	0	0	0	0	0	0	0	2	20	13.11	0.03
Tw	2	9	0	0	1	0	3	0	0	0	0	0	0	0	0	0	0	0	0	0	0	15	0.98	0.00
Ts	0	0	15	0	0	0	0	0	0	0	0	0	0	0	0	0	0	0	0	0	0	15	29.56	0.06
Gs	0	0	0	20	1	0	0	0	0	0	0	0	0	0	0	0	0	0	2	0	0	23	79.73	0.15
Fl	0	0	0	0	16	0	1	0	0	0	0	0	0	0	0	0	0	0	0	0	0	17	53.07	0.10
Bl1	0	0	0	0	0	24	0	0	0	0	0	0	0	0	0	0	0	0	1	0	0	25	16.59	0.03
Bl2	0	0	0	0	0	0	15	0	0	0	0	0	0	0	0	0	0	0	0	0	0	15	1.51	0.00
Rd	0	0	0	0	0	0	0	23	1	0	0	0	0	0	0	0	1	0	0	0	0	25	17.16	0.03
Sq	0	0	0	0	0	0	0	0	14	0	0	1	0	0	0	0	0	0	0	0	0	15	2.88	0.01
Ib1	0	0	0	0	0	0	0	0	0	15	0	0	0	0	0	0	0	0	0	0	0	15	6.85	0.01
Ib2	0	0	0	0	0	0	0	0	0	0	15	0	0	0	0	0	0	0	0	0	0	15	2.37	0.00
Ib3	0	0	0	0	0	0	0	0	0	0	1	14	0	0	0	0	0	0	0	0	0	15	2.05	0.00
Ib4	0	0	0	0	0	0	1	1	0	0	0	0	12	0	0	0	0	0	1	0	0	15	2.01	0.00
Ib5	0	0	0	0	0	0	0	0	0	0	0	0	0	13	0	0	0	2	0	0	0	15	0.29	0.00
Rb1	0	0	0	0	0	0	0	0	0	0	0	0	0	0	38	0	0	0	2	0	0	40	33.49	0.06
Rb2	0	0	0	0	0	0	2	0	0	0	1	0	0	0	0	11	0	0	1	0	0	15	5.54	0.01
Rb3	1	0	0	0	0	0	0	1	0	0	0	0	0	0	3	1	19	0	2	0	0	27	19.77	0.04
Rb4	0	0	0	1	0	0	0	0	0	2	0	0	0	1	0	0	0	11	0	0	0	15	8.94	0.02
Rb5	0	0	0	3	1	1	0	3	0	0	0	0	0	1	0	0	0	0	34	0	1	44	219.32	0.42
Pg	0	0	0	0	0	1	0	0	0	0	0	0	0	0	0	1	0	0	1	12	0	15	1.14	0.00
Sd	0	0	0	0	0	0	1	0	0	0	0	0	0	0	0	0	0	0	1	1	12	15	6.81	0.01
Tt	21	9	15	24	19	26	23	28	15	17	17	15	12	15	41	13	20	13	45	13	15	416	523.16	
P	0.86	1.00	1.00	0.83	0.84	0.92	0.65	0.82	0.93	0.88	0.88	0.93	1.00	0.87	0.93	0.85	0.95	0.85	0.76	0.92	0.80	0.86		
U	0.90	0.60	1.00	0.87	0.94	0.96	1.00	0.92	0.93	1.00	1.00	0.93	0.80	0.87	0.95	0.73	0.70	0.73	0.77	0.80	0.80	0.90		
T	Overall Accuracy 0.87																								

Notes: Cw: Clear water; Tw: Turbid water; Ts: Trees and shrubs; Gs: Grass;Fl: Farm lands; Bl: Bare lands; Rd: roads; Sq: squares;Ib: Industrial buildings; Rb: Residential buildings; Pg: Playgrounds;Sd: Shadow; Tt: Totals; TA: Total class area.

**Table A2:** Error matrix populated by estimated proportion of area for ULULC layer 3.

S	Cw	Tw	Ts	Gs	Fl	Bl1	Bl2	Rd	Sq	Ib1	Ib2	Ib3	Ib4	Ib5	Rb1	Rb2	Rb3	Rb4	Rb5	Pg	Sd	Wi
Cw	0.02	-	-	-	-	-	-	-	-	-	-	-	-	-	-	-	-	-	-	-	0.00	0.03
Tw	0.00	0.00	-	-	0.00	-	0.00	-	-	-	-	-	-	-	-	-	-	-	-	-	-	0.00
Ts	-	-	0.06	-	-	-	-	-	-	-	-	-	-	-	-	-	-	-	-	-	-	0.06
Gs	-	-	-	0.13	0.01	-	-	-	-	-	-	-	-	-	-	-	-	-	0.01	-	-	0.15
Fl	-	-	-	-	0.10	-	0.01	-	-	-	-	-	-	-	-	-	-	-	-	-	-	0.10
Bl1	-	-	-	-	-	0.03	-	-	-	-	-	-	-	-	-	-	-	-	0.00	-	-	0.03
Bl2	-	-	-	-	-	-	0.00	-	-	-	-	-	-	-	-	-	-	-	-	-	-	0.00
Rd	-	-	-	-	-	-	-	0.03	0.00	-	-	-	-	-	-	-	0.00	-	-	-	-	0.03
Sq	-	-	-	-	-	-	-	-	0.01	-	-	0.00	-	-	-	-	-	-	-	-	-	0.01
Ib1	-	-	-	-	-	-	-	-	-	0.01	-	-	-	-	-	-	-	-	-	-	-	0.01
Ib2	-	-	-	-	-	-	-	-	-	-	0.00	-	-	-	-	-	-	-	-	-	-	0.00
Ib3	-	-	-	-	-	-	-	-	-	-	0.00	0.00	-	-	-	-	-	-	-	-	-	0.00
Ib4	-	-	-	-	-	-	0.00	0.00	-	-	-	-	0.00	-	-	-	-	-	0.00	-	-	0.00
Ib5	-	-	-	-	-	-	-	-	-	-	-	-	-	0.00	-	-	-	0.00	-	-	-	0.00
Rb1	-	-	-	-	-	-	-	-	-	-	-	-	-	-	0.06	-	-	-	0.00	-	-	0.06
Rb2	-	-	-	-	-	-	0.00	-	-	-	0.00	-	-	-	-	0.01	-	-	0.00	-	-	0.01
Rb3	0.00	-	-	-	-	-	-	0.00	-	-	-	-	-	-	0.00	0.00	0.03	-	0.00	-	-	0.04
Rb4	-	-	-	0.00	-	-	-	-	-	0.00	-	-	-	0.00	-	-	-	0.01	-	-	-	0.02
Rb5	-	-	-	0.03	0.01	0.01	-	0.03	-	-	-	-	-	0.01	-	-	-	-	0.32	-	0.01	0.42
Pg	-	-	-	-	-	0.00	-	-	-	-	-	-	-	-	-	0.00	-	-	0.00	0.00	-	0.00
Sd	-	-	-	-	-	-	0.00	-	-	-	-	-	-	-	-	-	-	-	0.00	0.00	0.01	0.01
Tt	0.02	0.00	0.06	0.16	0.11	0.04	0.01	0.06	0.01	0.02	0.01	0.00	0.00	0.01	0.07	0.01	0.03	0.01	0.35	0.00	0.02	
